# Ageing and Parkinson's disease: Why is advancing age the biggest risk factor?^☆^

**DOI:** 10.1016/j.arr.2014.01.004

**Published:** 2014-03

**Authors:** Amy Reeve, Eve Simcox, Doug Turnbull

**Affiliations:** a Newcastle University Centre for Brain Ageing and Vitality, Institute for Ageing and Health, Newcastle University, Newcastle upon Tyne NE2 4HH, UK; b Wellcome Trust Centre for Mitochondrial Research, Institute for Ageing and Health, Newcastle University, Newcastle upon Tyne NE2 4HH, UK

**Keywords:** Parkinson's disease, Mitochondria, Ageing, Neurodegeneration

## Abstract

As the second most common age related neurodegenerative disease after Alzheimer's disease, the health, social and economic impact resulting from Parkinson's disease will continue to increase alongside the longevity of the population. Ageing remains the biggest risk factor for developing idiopathic Parkinson's disease. Although research into the mechanisms leading to cell death in Parkinson's disease has shed light on many aspects of the pathogenesis of this disorder, we still cannot answer the fundamental question, what specific age related factors predispose some individuals to develop this common neurodegenerative disease. In this review we focus specifically on the neuronal population associated with the motor symptoms of Parkinson's disease, the dopaminergic neurons of the substantia nigra, and try to understand how ageing puts these neurons at risk to the extent that a slight change in protein metabolism or mitochondrial function can push the cells over the edge leading to catastrophic cell death and many of the symptoms seen in Parkinson's disease. We review the evidence that ageing is important for the development of Parkinson's disease and how age related decline leads to the loss of neurons within this disease, before describing exactly how advancing age may lead to substantia nigra neuronal loss and Parkinson's disease in some individuals.

## Highlights

• Review of age related processes occurring within substantia nigra neurons.
• Discussion of why these neurons seem to be susceptible to loss with age.
• Review of why SN neurons are particularly sensitive to mitochondrial dysfunction.
• Review of why SN neurons are sensitive to changes in protein degradation pathways.
• Discussion of relevance to Parkinson's disease pathology.

## Graphical abstract

**Figure:**
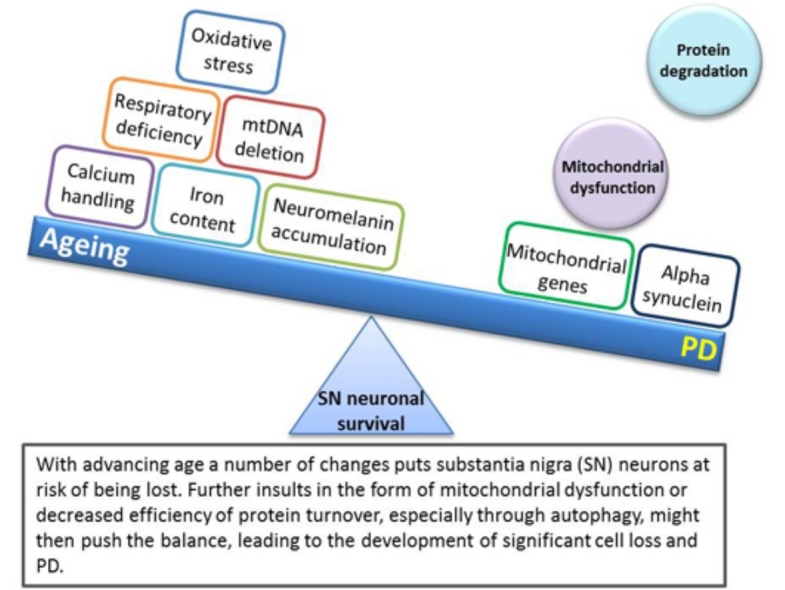

## 1 Introduction

Parkinson's disease (PD) affects over 1% of the population over the age of 60, which in the UK equates to over 127,000 individuals (or 500,000 individuals in the USA), while in individuals over the age of 85 this prevalence reaches 5%, highlighting the impact that advancing age has on the risk of developing this condition (de Lau and Breteler, 2006; Nussbaum and Ellis, 2003; Wood-Kaczmar et al., 2006). Although thought of as a disease of old age, a small percentage of patients (about 5% of all cases) present with symptoms before the age of 60 years and the majority of these cases are caused by mutations in an ever increasing list of genes which affect either protein metabolism or mitochondrial function, including Pink1 (PARK6), Parkin (PARK 2), DJ-1 (PARK7) and alpha-synuclein (PARK 1), thus highlighting that dysfunction in either is sufficient to cause PD (Gasser et al., 2011). These familial, early onset forms of Parkinson's disease can be autosomal dominant or recessive and may show a similar phenotype to sporadic PD in terms of symptoms and response to L-Dopa, but also may present with different symptoms and neuropathology.

**Fig. 1:**
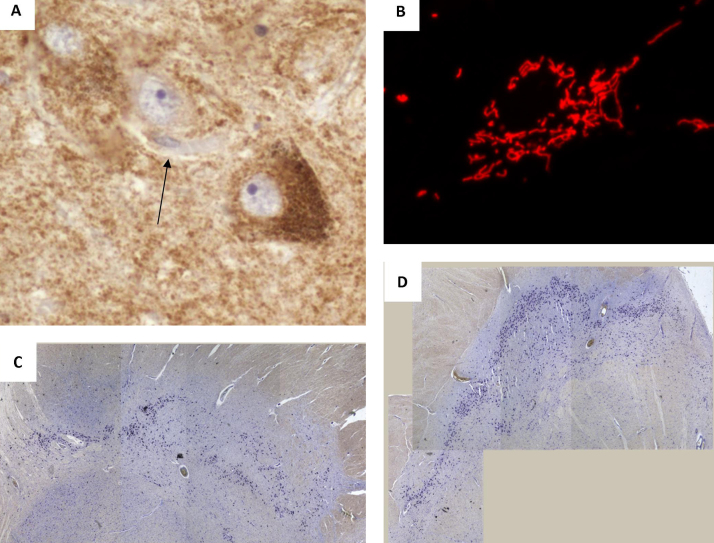
Changes within SN neurons with advancing age. (A) There is an increase in the number of cells showing mitochondrial dysfunction, including a loss of key mitochondrial proteins including complex I subunits (arrow). Changes in mitochondrial membrane potential and network dyanmics have also been shown to be important for neuronal survival image B, shows the mitochondrial network of a healthy neuron within culture, fragmentation of this network is associated with changes in mitochondrial membrane potential and prior to degradation through mitophagy. There is a loss of SN neurons with advancing age, images C and D show the SN of a 69 year old and a 53 year old respectively. The loss of neurons can be seen as a loss of pigmented cells even at a low magnification in the SN of the 69 year old.

Whilst Parkinson's disease involves a complex array of symptoms, an important brain region affected by severe cell loss in PD, and the main cause of the motor symptoms associated with this disorder, is the substantia nigra (SN). Specifically within the SN it is the dopaminergic neurons of the pars compacta that are lost and interestingly, this brain region also shows more pathological changes with normal ageing than any other region (Fig. 1). A recent study of over 750 elderly individuals (mean age 88.5 years) without clinically defined PD, has shown that nearly 1/3 showed mild to severe neuronal loss within the substantia nigra, with 10% also showing Lewy body pathology (Buchman et al., 2012). Cell loss within the SN has been shown to be extensive (Rudow et al., 2008) and was estimated to occur at a rate of 4.7% per decade (Fearnley and Lees, 1991), while more recent stereological techniques estimate this loss of neurons to occur at a rate of 9.8% per decade (Ma et al., 1999b).

Many studies have measured neuronal loss in other brain regions with ageing, although none have shown any degree of cell loss similar to that seen within the SN. Neuronal numbers have been shown to remain relatively stable throughout old age within the hippocampus, putamen, medial mammillary nucleus, hypothalamus and the nucleus basalis of Meynert, while it was estimated that the neocortical neurons might show a loss of only 10% over the entire lifespan (Pakkenberg and Gundersen (1997) and reviewed in Lowe (2008)). However, in other dopaminergic populations including in the ventral tegmental area and the retrorubral area, cell loss might actually reach 50% (Hirsch et al., 1987). These studies show that dopaminergic neuronal populations seem preferentially vulnerable to loss with ageing compared to many other brain regions and those related to other neurodegenerative disorders e.g. the hippocampus.

An understanding of why these SN cells die with advancing age may yield important insight into why cells are lost in PD. We review the literature to define how increasing age affects the neurons of the SN, why these cells in particular are lost and show dysfunction with advancing age, particularly in PD.

## 2 The environment of the substantia nigra

The neurons of the SN are distinct in many ways. They are pigmented, show pacemaking activity and increased oxidative stress related to the metabolism of dopamine within them. They are also thought to be particularly susceptible to the mitochondrial dysfunction which accumulates within them with advancing age (Bender et al., 2006; Kraytsberg et al., 2006). Here we consider the characteristics of SN neurons which may predispose them to an increased sensitivity to mitochondrial dysfunction and changes in protein degradation pathways, which may in turn be key to the pathogenesis of Parkinson's disease.

### 2.1 Dopamine metabolism and oxidative stress

Although a significant amount of oxidative stress is generated by the production of reactive oxygen species within mitochondria, SN neurons are believed to be under additional oxidative stress due to the metabolism of dopamine within them. Dopamine metabolism by monoamine oxidase generates a number of oxidative species including oxygen radicals and H_2_O_2_, while the oxidation of dopamine itself might occur through a number of processes including the presence of transition metals (reviewed in (Sulzer, 2007)). The main protector of dopaminergic neurons against this oxidative stress is the dopamine transporter (DAT). DAT takes the damaging dopamine back into the nerve terminal where it can then be repackaged into synaptic vesicles by VMAT2 (vesicular monoamine transporter 2). There have been reports that DAT expression declines with age in the dorsal tier of the SN but that DAT staining is greatest in the ventral tier, suggesting that perhaps this can account for some of the susceptibility of these neurons to loss in PD compared to the population within the dorsal tier (Ma et al., 1999a).

### 2.2 Calcium dynamics and pacemaking activity

Recent studies have implicated that the calcium handling of substantia nigra neurons and the importance of calcium for their firing activity are key to the pathogenesis of Parkinson's disease but also are an avenue through which to achieve neuroprotection (Chan et al., 2007). The neurons of the SN exhibit autonomous pacemaking activity, believed to be important for the maintenance of dopamine levels within the striatum. In adult neurons this activity is maintained by specific calcium channels (CaV1.3L-type) which have recently been shown to be prevalent in other neuronal populations which are vulnerable in PD (Goldberg et al., 2012). However, in juvenile neurons this activity is maintained by sodium channels and a reversion to juvenile forms of activity can be induced in adult neurons by blockage of the calcium channels by Isradipine. Interestingly, this reversion also protects the SN neurons against treatment with the mitochondrial toxin rotenone, known to cause specific SN neuronal loss and PD-like symptoms in model systems (Chan et al., 2007).

The way in which these neurons handle calcium and the calcium concentration within them is hugely important and it's sequestration within mitochondria is key for cellular survival. Mitochondria are responsible for the modulation of calcium within neurons to maintain intracellular levels, particularly important for the maintenance of neuronal excitability. In fact, studies in neurons differentiated from transmitochondrial cybrids have shown that neurons showing mitochondrial dysfunction show altered responses to multiple stimuli which leads to a prolonged elevation of intracellular calcium (Trevelyan et al., 2010). Changes in the ATP levels within neurons such as those that may occur in response to accumulating mitochondrial DNA mutations would affect the extrusion of calcium. This may lead to an overload of calcium within the neurons which in turn would lead to the mitochondrial permeability transition which then causes a loss of mitochondrial bioenergetic function. These events might also be initiated by oxidative stress which as discussed previously may occur for a number of reasons with advancing age within the neurons of the SN.

### 2.3 Iron concentration and changes

The iron content of substantia nigra neurons is thought to be an important contributor to their susceptibility to loss in PD, particularly based on the generation of reactive oxygen species by the Fenton reaction. Several studies, using various imaging techniques to assess how the iron content of the SN and other deep brain nuclei changes with advancing age, have shown that there is an increase in the iron content of the SN with advancing age and that a significant increase occurs above the age of 40 within the SN pars compacta (Bilgic et al., 2012; Daugherty and Raz, 2013; Dexter et al., 1989; Haacke et al., 2010; Sofic et al., 1991). However this data remains inconclusive since other studies have reported no change in the iron content of these neurons in PD compared to controls (reviewed in Friedman et al. (2007)). This does not mean that the iron content of these neurons is not important. It links ageing to a potentially detrimental characteristic of these neurons, and since neuromelanin (see below) is believed to bind iron and chelate it, again gives a plausible link between two important features of these neurons. The affinity of iron for melanin is much lower than for iron binding proteins such as ferritin, however it has been shown that neuromelanin does have a higher affinity for iron than other melanins (Double et al., 2003; Shima et al., 1997). The mitochondria, in particular a number of electron transport chain proteins, including complex I, rely on iron sulphur clusters for their function. Therefore changes in the concentration or availability of iron within these neurons will additionally impact on mitochondrial function and could thus exacerbate any mitochondrial dysfunction further, leading to a loss of these neurons.

### 2.4 Neuromelanin accumulation

The pigmentation of substantia nigra is due to the accumulation of neuromelanin within them. This pigment accumulates with advancing age, is composed of many different proteins and molecules and may afford SN neurons with some protection against oxidative stress. Neuromelanin begins accumulating from the 3rd year of life and shows a progressive increase with age (Fedorow et al., 2006; Halliday et al., 2005). It is composed of mainly lipids but also proteins and products from the metabolism of dopamine, explaining its distribution within very select populations of catecholamine neurons within the brain (reviewed in Double et al. (2008)). Neuromelanin is also found within other catecholaminergic populations, including the neurons of the locus coeruleus, ventral tegmental area and hypothalamus (reviewed in Double et al. (2008)). Although this pigment accumulates within all individuals with advancing age it has been implicated to be important for cell survival and in PD, for the vulnerability of dopaminergic neurons. Neuromelanin has been proposed to be a free radical scavenger, a regulator of intracellular iron and an inactivator of cellular cations. Therefore it is not surprising that changes in neuromelanin have been implicated to be important, contributing to the selective vulnerability of dopaminergic neurons in PD.

It has been shown that the ventral tier of the SN, which shows the most profound cell loss in PD, contains less neuromelanin than the somewhat more preserved dorsal tier, suggesting that this pigment might have a protective effect within these neurons (Gibb and Lees, 1991). Other studies have also suggested that the pigmented neurons of the SN contain less neuromelanin in PD brains than in the brain of healthy controls, again suggesting that this pigment might protect these neurons against intracellular stressors (Kastner et al., 1992; Zecca et al., 2001).

## 3 Mitochondrial dysfunction within substantia nigra neurons

The effect of mitochondrial dysfunction on the health and survival of neurons within the SN has been debated for over 3 decades, and although undoubtedly a contributor to the pathogenesis of PD and the loss of these neurons, the exact role that this organelle plays is yet to be elucidated.

**Fig. 2:**
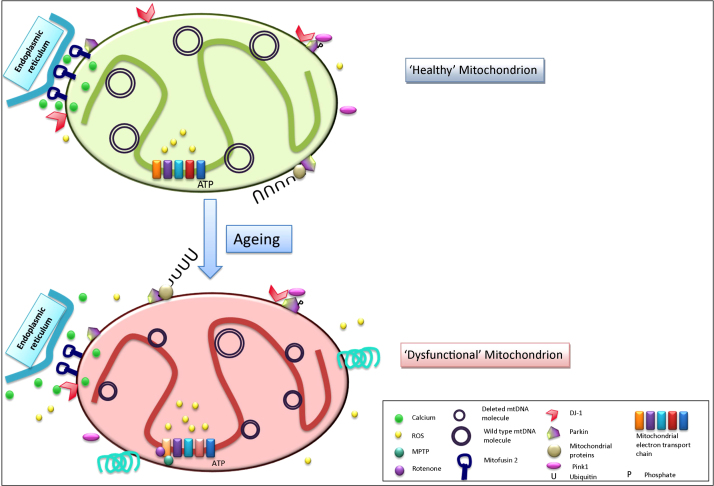
Mitochondrial dysfunction has been implicated to be important for the pathogenesis of Parkinson's disease for nearly 30 years. A number of different aspects of mitochondrial biology have been linked to PD. This figure shows how a healthy mitochondrion (green) changes with advancing age, for example accumulating mitochondrial DNA deletions (shown as smaller mtDNA molecules), and becomes dysfunctional (red) and highlights key proteins and processes that have been implicated in the pathogenesis of PD. Mitochondrial DNA deletions lead to mitochondrial dysfunction and respiratory deficiencies and have been linked to the generation of ROS (yellow) and the associated oxidative stress. Changes in the expression and activity of mitochondrial electron transport chain complexes I and IV have been found in the elderly and patients with PD and inhibition of complex I causes PD like symptoms in model systems treated with toxins such as MPTP (blue/green) and rotenone (purple). The association of the mitochondria with ER has been shown to be important for mitochondrial calcium handling (green) and this association relies on mitofusin 2 and DJ-1. The buffering of calcium by the mitochondria is important for the maintenance of cellular homeostasis. Alpha-synuclein, which forms Lewy bodies, has also been shown to interact with mitochondria and affect their function. Finally proteins encoded by a number of genes known to be mutated in autosomal recessive forms of PD have functions important for mitochondrial function as well as the targeting of mitochondria to mitophagy. Once phosphorylated by Pink1 (indicated by ‘P’), Parkin ubiquinates a number of mitochondrial proteins to target the mitochondria for degradation. These processes will all affect the survival of neurons in the SN with advancing age.

Mitochondrial dysfunction has been implicated to be important for the pathogenesis of Parkinson's disease since the discovery that MPP+ causes rapid parkinsonism and SN cell loss through inhibition of complex I of the electron transport chain (Langston et al., 1983; Langston and Ballard, 1983). This discovery was followed by reports of decreased complex I activity and protein expression in tissues from patients with PD (Schapira et al., 1989, 1990). The neurons of the SN are thought to be particularly susceptible to dysfunction within the mitochondria and support for a role for mitochondria, and specifically their dysfunction in the loss of SN neurons in PD, has been increasing over recent years. This section reviews the data to support how an age related decline in mitochondrial function will affect cellular function and survival. Mitochondrial dysfunction is likely to be a key player in the loss of these neurons based on its impact on neuronal processes and function (reviewed in Fig. 2).

### 3.1 Respiratory deficiency

The specific inhibition of key mitochondrial proteins including complex I has been known to cause Parkinsonian like symptoms since its inhibitors, MPP^+^ and rotenone, were shown to cause loss of SN neurons in both man and model systems (Betarbet et al., 2000; Langston et al., 1983). Respiratory deficiency can be defined as a decline in the activity of complex IV which can be visualised in tissue using the COX/SDH assay. Bender et al. report that approximately 3% of SN neurons are COX deficient in patients with PD compared to 1% in age matched controls, while other studies have reported up to 30% COX deficiency in some cases (Bender et al., 2006; Itoh et al., 1996; Kraytsberg et al., 2006). Furthermore, Elson et al. predicted that 1–4% of post mitotic cells in 80–120 year olds would be COX deficient and that this accumulation would begin from the age of 60 (Elson et al., 2001). The development of respiratory deficiency within the neurons of the SN, may also lead to compromised production of ATP. Therefore considering recent modelling data suggesting that the propagation of the action potential within SN neurons is highly energy dependent based on the complexity of the axonal arbour of these neurons, a decrease in the production of ATP will affect the excitability of these neurons which would further increase their vulnerability (Pissadaki and Bolam, 2013).

#### 3.1.1 mtDNA mutation load

The respiratory (COX) deficiency detected within the SN has been shown to be caused specifically by mitochondrial DNA deletions and these deletions were found to reach levels of 50% (Bender et al., 2006; Kraytsberg et al., 2006). This deletion load is higher than in other brain regions and tissues where respiratory deficiency is below 15% with even advanced age and unlike some other aged tissues there is no accumulation of mtDNA point mutations within the SN (Reeve et al., 2009; Taylor et al., 2003). Several studies have compared the mtDNA deletion load between other brain regions and the SN in both ageing and PD. Deletion levels have been found to be higher in the SN than in the locus coeruleus, ventral tegmental area, frontal cortex and the putamen (Bender et al., 2008; Elstner et al., 2011). While in 1992 two studies investigated the level of mtDNA deletions within 12 different brain regions with advancing age. They found that in many of the brain regions studied the level of the ‘common’ mtDNA deletion accumulated with advancing age but that the levels of this mutation were highest in the SN followed by the basal ganglia, cortical areas and the cerebellum (Corral-Debrinski et al., 1992; Soong et al., 1992). Respiratory deficiency due to high mtDNA deletion levels is however found in other neuronal populations in patients with Alzheimer's disease (AD) and multiple sclerosis (Campbell et al., 2011; Krishnan et al., 2012). The level of mtDNA mutation and respiratory deficiency has been shown to correlate with the cell loss seen within the cerebellum of patients affected by ataxia as part of their mitochondrial disease (Lax et al., 2012). This data therefore would suggest that if the neurons of the SN are particularly susceptible to the mitochondrial dysfunction shown to cause cell loss elsewhere in the brain, then it is likely that this dysfunction is also associated with the cell loss seen in PD.

Although there is no definitive evidence as yet to support a mechanism for the formation of these deletions, we have hypothesised that they could be formed through the repair of damage to mitochondrial DNA (Krishnan et al., 2008). This damage leads to the formation of double strand breaks which are then repaired leading to the loss of several kilobases of the mitochondrial genome. This damage could be due to a number of the processes described above, but is likely to reflect the highly oxidative environment of the SN. This theory has been strengthened by the work of Pickrell et al. who specifically targeted a mitochondrial restriction enzyme, *Pst1*, to the neurons of the SN. They showed that the induction of expression of this enzyme led specifically to the formation of double strand breaks within the mtDNA leading to deletion formation and depletion, and importantly the development of motor symptoms and SN neurodegeneration (Pickrell et al., 2011). Therefore the formation of these mutations within human SN neurons is likely to be a consequence of the processes mentioned above. They then lead to reduced mitochondrial function, ATP levels and eventually cell death. In addition mitochondrial DNA deletions have previously been shown to cause a reduction in proteasomal activity which could exacerbate the accumulation of misfolded protein, in particular alpha-synuclein, as discussed below (Alemi et al., 2007).

Disruption of mitochondrial DNA integrity in mice causes an ageing phenotype and the loss of dopaminergic neurons (Ekstrand et al., 2007; Trifunovic et al., 2004). Initially, mice were created with a proof reading deficient knock-in of the mtDNA polymerase, POLG. These mice developed a premature ageing phenotype including kyphosis, weight loss and osteoporosis, caused by an increase in somatic mtDNA mutations, including both point mutations and deletions (Trifunovic et al., 2004). This study highlighted the importance of mitochondrial DNA for ageing and hinted that the accumulation of defects within this genome could lead to many of the age related changes that occur. Furthermore, a subsequent study by the same group showed that the conditional knock-out of the mitochondrial transcription factor A, Tfam, specifically within dopaminergic neurons causes a reduction in mtDNA expression and importantly progressive parkinsonism within the mice, dopaminergic neuron loss and the accumulation of protein inclusions (Ekstrand et al., 2007). The expression of mutated Twinkle in dopaminergic neurons has shown the importance of mtDNA deletions in the survival of SN neurons and also highlighted changes in the expression of parkin, an important protein in the mitochondrial degradation pathway. Song et al. showed that in older mutant mice there was a specific loss of SN neurons which correlated with the development of motor defects, and that the development of the SN cell loss was related to the accumulation of mtDNA deletions. Interestingly, mutant Twinkle causes a reduction in the expression of parkin within the midbrain and decreased proteasomal activity (Song et al., 2012). This evidence highlights that disruptions of mtDNA within dopaminergic neurons are sufficient to cause the symptoms and neuropathology associate with PD and thus the importance of the accumulation of mitochondrial dysfunction with advancing age to the probable pathogenesis of this disease.

#### 3.1.2 Disruption of key mitochondrial processes

As the number of genes in which mutations are associated with PD increases so too does the evidence that changes in mitochondrial dynamics and turnover are important for the loss of neurons within the SN. The knockout of alpha-synuclein, pink1, parkin and DJ-1 in *drosophila* and cell culture based models leads to alterations in mitochondrial morphology and network formation (Clark et al., 2006; Greene et al., 2003; Martin et al., 2006; Nakamura et al., 2011; Park et al., 2005, 2009). Mutations in alpha-synuclein have been shown to cause fragmentation of the mitochondrial network and changes in the ultrastructure and distribution of the mitochondria suggesting increased mitochondrial fission, (Nakamura et al., 2011). Mutations in Pink1 and Parkin have been shown to cause a loss of mitochondrial ultrastructure as observed as changes in the electron density of the mitochondria and fragmentation of the mitochondrial network (Clark et al., 2006; Greene et al., 2003). A loss of DJ-1 causes a loss of mitochondrial membrane potential and increased mitochondrial fragmentation (Thomas et al., 2010; Wang et al., 2012) and mitochondrial changes have also been reported when LRRK2 (Leucine rich repeat kinase 2) is mutated (Goo et al., 2013; Mortiboys et al., 2010).

A recent paper has also shown that mitofusin2 (mfn2), a protein important for mitochondrial fusion, is essential for the survival of striatal projections from the SN. Indeed knockout of mfn2 has no effect on the number of dopaminergic SN neurons but causes a dramatic reduction in the number of dopaminergic nerve terminals in the striatum of affected mice, an accompanying reduction in striatal dopamine levels and locomotor disturbances (Lee et al., 2012). Mfn 2 is also a key protein for the handling of calcium within the mitochondria, the ability of mitochondria to take up calcium is reliant on their close proximity to the endoplasmic reticulum (ER) and therefore the site of calcium release. The proximity of the mitochondria to the ER is reliant on mfn2 which is enriched at the contact sites between the ER and mitochondria (de Brito and Scorrano, 2008). Recent evidence has also linked the expression of DJ-1 and parkin to this interaction, DJ-1 modulates the interaction of the mitochondria and the ER and in doing show suppresses the effects of p53 on mitochondrial calcium handling and morphology (Ottolini et al., 2013). Interestingly, parkin overexpression has been shown to also enhance the calcium handling capabilities of the mitochondria by enhancing the interaction of the mitochondria with the ER without a subsequent effect on the mitochondrial calcium uptake machinery (Cali et al., 2012). The neurons of the SN, as mentioned above, use calcium to maintain their pacemaking activity and therefore changes in how the neurons handle and maintain calcium levels are going to be hugely influential on both the activity and survival of these neurons.

Both fission/fusion and mitochondrial movement require intact mitochondrial membrane potential (ΔΨm) and a loss of this potential is associated with mitochondrial degradation through mitophagy (Twig et al., 2008). Subtle differences in ΔΨm have also been attributed to determining directionality of neuronal mitochondria, for example 80% of mitochondria with a low membrane potential are transported towards the cell body (Miller and Sheetz, 2004), although this might not be such a clear cut distinction (Verburg and Hollenbeck, 2008). Studies using transmitochondrial cybrids have shown that neurons with mtDNA mutations causing a severe mitochondrial complex I defect have a significantly increased ΔΨm, due to reversal of the ATP synthase, however mutations in complex IV seemed to not affect the ΔΨm (Abramov et al., 2010). The effect of these mutations on the ΔΨm in these cells is interesting considering the effect on mitochondrial protein function and expression of the accumulating mtDNA defects we see in aged SN neurons. It has been proposed by a number of studies that the degradation of mitochondria through the specific autophagy pathway, mitophagy, is heavily reliant on the dissipation of ΔΨm, followed by fission from the mitochondrial network and loss of OPA1 expression (Twig et al., 2008). Alterations in mitochondrial transport have been implicated to be important in a number of neurodegenerative conditions including Alzheimer's disease and Huntington's disease (Reddy and Shirendeb, 2012; Reddy et al., 2012). In Parkinson's disease, alterations in mitochondrial transport occur in response to rotenone treatment and pink1 has been shown to interact with Milton and Miro, two key proteins responsible for the interaction of mitochondria with neuronal motor proteins (Arnold et al., 2011; Weihofen et al., 2009). Therefore if mitochondria which harbour defects of the electron transport chain do not lose their ΔΨm then they will not leave the network and would persist within the neuron, which might explain why neurons accumulate such a high proportion of dysfunctional mitochondria over time.

Parkin and pink1 have been implicated to be important for the targeting of mitochondria for degradation (discussed in detail below). Therefore the very fact that dysfunctional mitochondria persist within a neuron and accumulate to a level sufficient to cause a loss of complex IV activity and complex I protein expression would suggest that this pathway in particular shows an age related decline. Recent evidence suggests that the interaction of pink1 and parkin might actually facilitate the specific turnover of mitochondrial respiratory chain subunits (Vincow et al., 2013). These interactions might suggest a reason for the loss of key subunits of the electron transport chain, as the system might preferentially target these subunits, reducing the expression of these proteins, rather than degrading intact, yet dysfunctional mitochondria. Furthermore this might lead to the accumulation of misfolded mitochondrial proteins which would put further burden onto the protein degradation pathways, described below.

#### 3.1.3 Interaction with alpha synuclein

The pathological hallmark of PD and other synucleinopathies including dementia with Lewy bodies (DLB), is the Lewy body. The main protein component of these structures is alpha synuclein, although both the effect of these inclusions on neuronal survival and the toxicity of different forms of this protein are still debated, it seems likely that the oligomeric forms of alpha-synuclein are the most toxic (reviewed in Kalia et al. (2013)). Alpha-synuclein itself is a widely expressed protein, with an alpha helical structure (Bendor et al., 2013), and is believed to be important for the synaptic vesicle recycling (Cheng et al., 2011; Murphy et al., 2000). The exact mechanism that leads to the conformational change believed to be required for its aggregation in PD needs to be fully elucidated. However, what is clear is that Lewy bodies and neurites show a high β-sheet content and a distinctive cross-β X-ray diffraction pattern. This pattern is interestingly also observed for fibrillar deposits in other neurodegenerative disorders including Alzheimer's disease, and aggregated alpha-synuclein is strikingly similar to amyloid (Bendor et al., 2013; Chiti and Dobson, 2006). Alpha synuclein has also been found to aggregate in many cases of AD although interestingly predominantly within the amygdala rather than the SN (Leverenz et al., 2008; Uchikado et al., 2006). Lewy body pathology is also seen in elderly individuals with no Parkinson's disease symptoms and recently we found evidence of Lewy body pathology in a patient with POLG mutations, but with no corresponding extrapyramidal features (Reeve et al., 2013).

Many modifications have been proposed to be important for the aggregation of alpha-synuclein and its conformational change, importantly these include oxidation, the presence of heavy metals and phosphorylation (reviewed in Bendor et al. (2013)) (Breydo et al., 2012; Fujiwara et al., 2002; Giasson et al., 2000). Accumulating mtDNA mutations or dysfunctional mitochondria are likely to have an effect on the oxidative stress levels within SN neurons, which might contribute to the misfolding and accumulation of this protein. However, in a recent study we showed that Lewy body pathology was associated with neurons with intact expression of key mitochondrial electron transport chain proteins, including complex I, suggesting at least that intact mitochondrial function and hence ATP levels are required for the aggregation of this protein within the cell body, perhaps relating to the transportation of this protein (Reeve et al., 2012). Numerous studies have used rotenone and other toxins to induce mitochondrial dysfunction and monitor the accumulation of alpha-synuclein, despite the wealth of information that these studies provide they often do not reflect the subtleties of the slow accumulation of mitochondrial dysfunction within ageing substantia nigra neurons. Our data might suggest that normal mitochondrial function might protect against cell loss and that those cells with mitochondrial dysfunction and Lewy body pathology are lost, or that the accumulation of alpha-synuclein into Lewy bodies requires mitochondrial function and that this accumulation prevents toxic, damaging alpha-synuclein species from accumulating at synapses.

Alpha-synuclein itself has been shown to interact with mitochondria and be imported into the mitochondria in an energy dependant manner (Devi et al., 2008). The accumulation of alpha-synuclein within mitochondria has been shown to lead to complex I impairment, decreased ΔΨm and increased ROS production (Devi et al., 2008; Parihar et al., 2008, 2009). The effect of these changes is likely to be an exacerbation of the mitochondrial defect present in ageing SN neurons. Mutations in the alpha-synuclein gene have been shown to cause changes in mitochondrial structure particularly affecting the cristae in cell culture, while *drosophila* models have highlighted the contribution of oxidative stress to the neuronal changes, for example showing hypersensitivity to hyperoxia (Botella et al., 2008). Transgenic mice expressing mutant forms of alpha synuclein have also been generated but they often do not show SN dopaminergic neuronal loss, despite very severe phenotypes and neuron loss in other brain regions closely associated with inclusion formation and mitochondrial degeneration (Martin et al., 2006). Interestingly, this neurodegeneration was also associated with mtDNA damage and a loss of complex IV activity (Martin et al., 2006). It is worth noting that although such models may not show SN pathology they often show pathology in other regions, for example LB-like inclusions in the motor neurons of the spinal cord (Martin et al., 2006). However, there are a number of difficulties that arise when trying to model human disease in mice and this is especially true for the SN, when you consider the life span of mice and the fact that their SN neurons do not contain neuromelanin. But these studies do highlight the close relationship and importance for the development of neuronal loss of two age related phenomena, those of increased mtDNA damage and also protein accumulation.

#### 3.1.4 PD symptoms in patients with mitochondrial disease

If mitochondrial dysfunction is particularly important in the pathogenesis of PD then we might expect to see PD symptoms in patients with mitochondrial disorders and potentially at a younger age than in the case of sporadic PD. We have shown that changes within the SN, and in particular cell loss within this brain region, is more closely associated with mitochondrial defects that are acquired rather than those that are inherited (Reeve et al., 2013). While a recent study in the POLG *D257A* mouse have shown that mtDNA deletions were not associated with SN neuronal degeneration and actually induced neuroprotective changes including changes in the ultrastructure of the mitochondria and compensatory changes in ΔΨm (Perier et al., 2013). Although extrapyramidal features are relatively rare in patients with mitochondrial disorders there have been a number of reports of Parkinsonism associated with mutations within *POLG*. In these cases there is often pigmented SN neuron loss and on occasions Lewy body pathology (Betts-Henderson et al., 2009; Luoma et al., 2004) and however we have detected Lewy body pathology in a POLG patient with no discernible cell loss or PD-like symptoms (Reeve et al., 2013). The age of onset of PD symptoms in *POLG* patients seems to vary with many being within the range of sporadic PD (Betts-Henderson et al., 2009; Luoma et al., 2004), but some cases show an earlier onset (Davidzon et al., 2006; Hudson et al., 2007; Mancuso et al., 2004). Many of these earlier onset cases have a family history, but they do not report whether common PD genes were screened for mutations, therefore it is difficult to fully dissect whether the *POLG* mutation is causative of the PD-like symptoms. However, the incidence of these symptoms in patients with *POLG* mutations is higher than for other mitochondrial patients and abnormal DAT scans are often seen in these patients. If the *POLG* mutation was responsible for the parkinsonism then one might expect most *POLG* patients to exhibit this phenotype, many patients with *POLG* mutations have cell loss within the SN, but this is not always associated with PD like symptoms (Reeve et al., 2013). Aside from pathogenic mutations within the *POLG* gene, several studies have investigated the association with PD of changes within the poly-glutamine expansions within the *POLG1* gene in different populations (Anvret et al., 2010; Balafkan et al., 2012; Eerola et al., 2010). These studies have shown a trend towards an association with non10-11Q repeats and PD in populations from Norway, America (Caucasian) and Sweden (Anvret et al., 2010; Balafkan et al., 2012; Eerola et al., 2010). These cases highlight that mitochondrial dysfunction is detrimental for the survival of SN neurons and that the accumulation of mtDNA mutations affects their survival with advancing age. Although mutations in *POLG* are the most common to be associated with parkinsonism in mitochondrial disease patients, there are reports of point mutations associated with these symptoms (Casali et al., 2001; Horvath et al., 2007; Siciliano et al., 2001; Simon et al., 1999).

In addition, several studies have also investigated whether certain mtDNA haplogroups are associated with susceptibility for the development of PD. Many studies seem to agree that haplogroups J, K are protective against the development of Parkinson's disease within European populations, while a large study has also suggested that T and super-haplogroup JT are also protective (Hudson et al., 2013; Huerta et al., 2005; Latsoudis et al., 2008; Pyle et al., 2005; van der Walt et al., 2003). However in other populations these haplogroups are not associated with a decreased risk of developing PD (Mehta et al., 2009). Furthermore an increased risk of developing PD with advancing age has been associated with the superhaplogroup HV (Hudson et al., 2013). These data highlight that even subtle changes within the mitochondria may impact on the likelihood of developing PD and thus be important for the pathogenesis of this disease.

## 4 Impairment of protein degradation in substantia nigra neurons

**Fig. 3:**
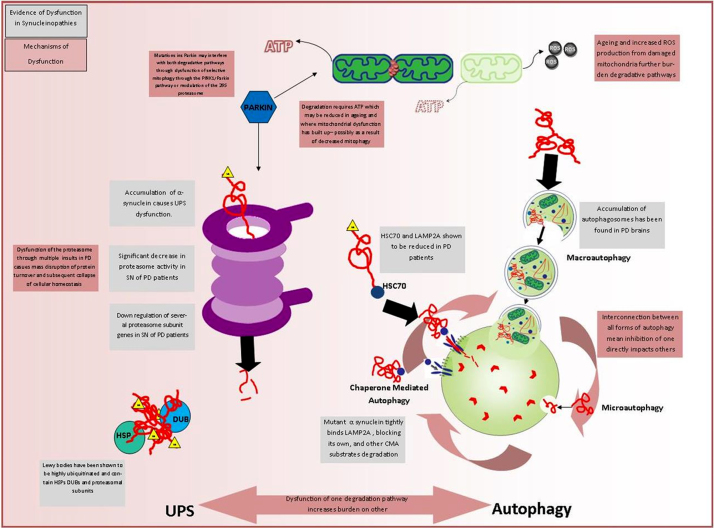
The degradation of proteins and organelles through the proteasome and autophagy pathways is tightly regulated and heavily ATP dependant. Both these processes have been implicated to be affected/dysregulated in Parkinson's disease. This figure reviews how these pathways are affected and the mechanism for their dysfunction. The interconnectivity between the two protein degradation pathways means that a decrease in the efficiency of one will strongly impact on the burden of the other. HSC70 – heat shock 70 kDa protein, DUB – deubiquitinating enzyme.

There are two main pathways within neurons for the degradation and removal of damaged proteins, the ubiquitin proteasome system (UPS) and autophagy, which encompasses the routes through which substrates are degraded through the lysosome (highlighted in Fig. 3). Both systems show a reduction in function and efficiency with age (Jana, 2012; Li and Li, 2011; Rubinsztein et al., 2011), are both linked to mitochondrial function since both require ATP and alterations in both have also been implicated in the development of both idiopathic and familial forms of PD. Although reductions in the efficiency of these pathways with ageing are likely to affect many populations, the SN neurons could still be predisposed to being more severely affected by changes in these processes.

The accumulation of damaged proteins in neurons with advancing age is related to oxidative adducts caused by reactive oxygen and nitrogen species which, as mentioned previously, accumulate within the SN with advancing age. Therefore the environment of the SN predisposes the neurons to be under significant oxidative stress with a high concentration of damaged proteins, thus efficient protein degradation pathways need to be in place to cope with an increased demand. Failure of such pathways could contribute to the accumulation of damaging proteins such as alpha-synuclein and could ultimately contribute to neuronal loss within this brain region.

### 4.1 The ubiquitin proteasome system

The ubiquitin proteasome pathway acts to remove soluble intracellular proteins. Briefly, the proteins to be degraded are covalently linked to ubiquitin chains, which target the protein to the proteasome, where it is unfolded and passes through the barrel of the 26S proteasome and the products of degradation are expelled at the end. Proteolysis through the UPS is a highly energy dependent process, requiring ATP at all stages from ubiquitination, through proteasome assembly, and final degradation and recycling of components. Because of this, the system is vulnerable to mitochondrial dysfunction, through reduction of energy provision and the direct inhibition of the proteasome through oxidised proteins and aggregations thereof. In ageing there is a qualitative reduction of the ubiquitin proteasome system (Emmanouilidou et al., 2010). In post mortem tissue, enzymatic reactions have shown that there is a reduction in proteasome activity in the SN of PD patients compared to age matched controls (McNaught et al., 2003; McNaught and Jenner, 2001). Moreover, mutations in Parkin known to cause an early onset, autosomal recessive form of PD is important for the targeting of proteins to the proteasome and expression of parkin proteins has been shown to be reduced in PD. Furthermore, inhibition of the proteasome has been shown to cause neurodegeneration and Lewy body like inclusions in a number of model systems while genetic ablation of subunits of the proteasome leads to extensive motor symptoms (Bedford et al., 2008). Conversely, mutant alpha synuclein can cause a direct inhibitory effect on the 20S cores proteolytic capabilities and the downregulation of several subunits of the proteasome in the substantia nigra of PD patients (Chen et al., 2005; Emmanouilidou et al., 2010; Tanaka et al., 2001).

### 4.2 Autophagy

Macroautophagy (hereafter autophagy) is a highly conserved process occurring in most organisms from yeast to man, responsible for the degradation of long lived proteins and organelles. This process requires the sequestration of the target protein within a double membrane, the autophagosome, the delivery of the autophagosome to the lysosome, fusion with the lysosome and the efficient degradation by lysosomal hydrolases. Although the main regulator of autophagy is the supply of nutrients to the cell, this dynamic process has been implicated to be important in ageing and a number of age related diseases, especially those involving the accumulation of protein aggregates within neurons. Knock-out of key autophagy related genes, ATG 5 and 7, has been shown to cause neurodegeneration in mice, particularly a loss of the Purkinje cells of the cerebellum (Hara et al., 2006; Komatsu et al., 2006).

The degradation of whole mitochondria occurs through the specific autophagy pathway, mitophagy. A number of genes that have been shown to cause familial autosomal-recessive forms of Parkinson's disease are known to be important for the targeting of mitochondria to this pathway, such as *Parkin* and *Pink1.* The recruitment of parkin, a ubiquitin E3 ligase, to damaged mitochondria requires the function of the putative kinase, Pink1. In addition, accumulation of autophagosomes has been found in PD brains reflecting the decrease in successful clearance in this disease (Anglade et al., 1997). This observation suggests that perhaps the autophagy pathway becomes overwhelmed, or that there is an over production of autophagosomes, perhaps in response to the accumulating dysfunctional mitochondria. Alternatively, an accumulation of these ‘end stage’ vesicles may actually represent a decrease in autophagy and autophagic flux (Ma et al., 2012; Zhou et al., 2012). The pathway by which mitochondria are degraded through autophagy is a tightly regulated process, which in theory should degrade dysfunctional mitochondria preventing their age associated accumulation. Recent mouse studies have shown that dysfunctional mitochondria do not necessarily recruit parkin and that in fact the absence of parkin may not affect the phenotype or clearance of mitochondria (Sterky et al., 2011). One of the other proposed substrates for Parkin is alpha-synuclein. Although usually degraded through the proteasome, alpha-synuclein requires unfolding for degradation and aggregating forms of this protein may require clearance through other pathways. Alpha-synuclein, however, is also a target for chaperone mediated autophagy, which through interactions with LAMP-2A and HSC (heat shock 70 kDa proteins) targets the alpha-synuclein directly to the lysosome (Alvarez-Erviti et al., 2010, 2013; Vogiatzi et al., 2008).

Clearly one of the most relevant relationships is that of the protein degradation pathways and their degradation of alpha-synuclein. This protein has been shown to be ubiquitinated for degradation through the proteasome, as well as degraded through chaperone mediated autophagy. Mutated forms of alpha-synuclein can bind to the lysosomal membrane without translocation through interactions with Lamp-2A, essentially blocking their own, and other substrates degradation through this pathway. Considering that the pathological hallmark of Parkinson's disease is the accumulation of Lewy body structures, it would seem only logical that the systems within the cell designed to remove these damaging forms of alpha-synuclein might show reduced efficiency. Similarly, the observation that neurons within the SN accumulate dysfunctional mitochondria would imply that with ageing the efficiency of mitophagy declines as damage to the mitochondria and mtDNA increases. Alongside these observations are investigations into changes in the expression of deubiquitinating enzymes (DUBs). DUBs act as a signal for both proteasomal and lysosomal degradation of proteins, and provide a scaffold for signal transduction. Their accumulation has been shown to occur within LBs suggesting that one cause of the accumulation of alpha-synuclein into these structures may be impaired autophagic flux (Tanaka et al., 2001).

## 5 Comparison with other neuronal populations that are vulnerable in PD

The neurons of the SN are not the only ones to be affected in PD (Braak et al., 2004; Del Tredici and Braak, 2012), do these other neuronal populations show any similarities with the neurons of the SN? Changes have been reported to also occur in several other brain stem nuclei including; the pedunculopontine nucleus (PPN), ventral tegmental area (VTA), the dorsal motor nucleus of the vagus nerve (DMV) and the locus coeruleus (LC). These neuronal populations show similarities with the neurons of the SN which may highlight not only their vulnerability in PD but also the processes key for the loss of SN neurons (Surmeier and Sulzer, 2013). The medium sized neurons of the LC, for example, contain neuromelanin, are noradrenaline containing, TH positive and show a loss of around 70% in PD (Gesi et al., 2000). These neurons show alterations in the ultrastructure of their synaptic mitochondria in PD, suggesting mitochondrial alterations might occur within these neurons (Baloyannis et al., 2006). There is also a reduction in the large cholinergic neurons of the PPN, 50% of these neurons have been reported to be lost in PD (reviewed in (Pahapill and Lozano, 2000)). Interestingly LB pathology has been found in these neurons in both Lewy body disease (LBD) and Alzheimer's disease, but prominent cell loss was only found in LBD (Dugger et al., 2012).

The cholinergic neurons of the DMV show Lewy body pathology in patients with PD at Braak stage 1, while pathology only appears in the SN at stage 3 (Braak et al., 2004). The vulnerability of this group of neurons in PD has recently been investigated. These neurons also rely on pacemaking activity, similar to the neurons of the SN. A major contributor to this activity within the neurons of the DMV are the L-type CaV1.3 channels and similar to the SN neurons DMV neurons show evidence of mitochondrial stress during pacemaking activity (Goldberg et al., 2012). Furthermore, the dopaminergic neurons of the VTA also show pacemaking activity, but the activity in these neurons is modulated primarily by sodium with minimal contribution from calcium channels (Khaliq and Bean, 2010). It is therefore interesting that in comparison to the neurons of the SN the VTA neurons are relatively spared, in addition the neurons of the VTA have been shown to be resistant to the overexpression of alpha-synuclein, known to cause the loss of SN neurons (Maingay et al., 2006). The development of LB pathology in a number of other brain stem nuclei has also been investigated and interestingly within this group of nuclei only those which show an accumulation of lipofuscin or neuromelanin show an increased likelihood of developing this pathology (Braak et al., 1995, 2001).

Therefore vulnerable neuronal populations show some characteristics which are common to those of SN neurons, suggesting that the unique mixture of these features within the SN contributes significantly to the development of PD.

## 6 Conclusion

**Fig. 4:**
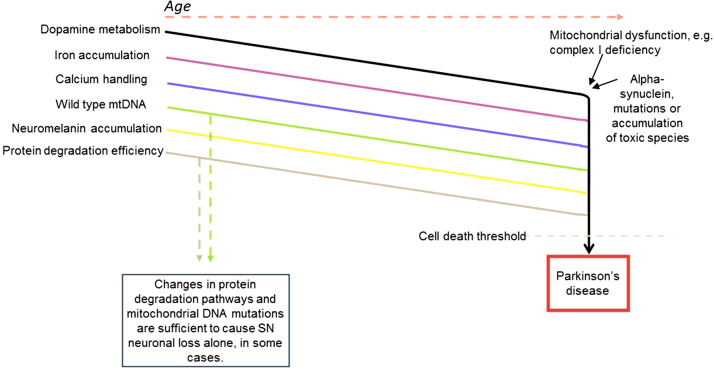
Age related changes in a number of processes pushes substantia nigra neurons towards cell death. These changes include accumulation of mitochondrial DNA defects, oxidative damage (through a number of processes) and accumulation of neuromelanin. This increases the vulnerability of the SN neurons so that a further insult from either toxic alpha-synuclein or mitochondrial dysfunction leads to cell death. It is the accumulation of all these processes that will lead to the loss of neurons within this brain region. Several of these processes have been shown to be sufficient to cause substantia nigra neuronal loss alone and so are likely to contribute to the death of these neurons in Parkinson's disease.

With advancing age a number of processes essential for the function of substantia nigra neurons including dopamine metabolism, wild type mitochondrial DNA copy number and protein degradation decline (Fig. 4). Dopamine metabolism generates a significant amount of reactive oxygen species that will affect a number of different processes within the neurons, a decline in wild type mtDNA copy number will lead to a decrease in ATP production and a reduction in efficient protein degradation will affect the functioning of neurons. In addition accumulation of neuromelanin, the ability of neurons and mitochondria to handle calcium and the levels of iron within these neurons will also be affected, so that additional insults such as mitochondrial complex I and IV deficiencies and aggregating alpha-synuclein causes the loss of vulnerable neurons, once this cell loss reaches a certain level, the symptoms of PD develop. Any minor changes that affect this vulnerability to accumulating damage may explain why not all individuals are affected by PD with advancing age. In isolation some of these processes including mitochondrial DNA mutations and changes in protein degradation pathways have been shown to cause neuronal loss (Hara et al., 2006; Komatsu et al., 2006; Reeve et al., 2013), but with advancing age it is the accumulation of many defects that renders the neurons of the SN vulnerable to the additional insults of mitochondrial deficiencies and toxic alpha-synuclein species.

Therefore, in conclusion we believe that ageing effects cause a cascade of stressors within the substantia nigra which essentially weakens the neurons and their ability to respond to further insults that are seen as part of the disease process. Further mitochondrial dysfunction and alterations in protein degradation pathways are subsequently far more detrimental to the neurons of the SN than they would be to neurons elsewhere within the brain.

## References

[bib0005] Abramov A.Y., Smulders-Srinivasan T.K., Kirby D.M., Acin-Perez R., Enriquez J.A., Lightowlers R.N., Duchen M.R., Turnbull D.M. (2010). Mechanism of neurodegeneration of neurons with mitochondrial DNA mutations. Brain.

[bib0010] Alemi M., Prigione A., Wong A., Schoenfeld R., DiMauro S., Hirano M., Taroni F., Cortopassi G. (2007). Mitochondrial DNA deletions inhibit proteasomal activity and stimulate an autophagic transcript. Free Radic. Biol. Med..

[bib0015] Alvarez-Erviti L., Rodriguez-Oroz M.C., Cooper J.M., Caballero C., Ferrer I., Obeso J.A., Schapira A.H. (2010). Chaperone-mediated autophagy markers in Parkinson disease brains. Arch. Neurol..

[bib0020] Alvarez-Erviti L., Seow Y., Schapira A.H., Rodriguez-Oroz M.C., Obeso J.A., Cooper J.M. (2013). Influence of microRNA deregulation on chaperone-mediated autophagy and alpha-synuclein pathology in Parkinson's disease. Cell Death Dis..

[bib0025] Anglade P., Vyas S., Javoy-Agid F., Herrero M.T., Michel P.P., Marquez J., Mouatt-Prigent A., Ruberg M., Hirsch E.C., Agid Y. (1997). Apoptosis and autophagy in nigral neurons of patients with Parkinson's disease. Histol. Histopathol..

[bib0030] Anvret A., Westerlund M., Sydow O., Willows T., Lind C., Galter D., Belin A.C. (2010). Variations of the CAG trinucleotide repeat in DNA polymerase gamma (POLG1) is associated with Parkinson's disease in Sweden. Neurosci. Lett..

[bib0035] Arnold B., Cassady S.J., VanLaar V.S., Berman S.B. (2011). Integrating multiple aspects of mitochondrial dynamics in neurons: age-related differences and dynamic changes in a chronic rotenone model. Neurobiol. Dis..

[bib0040] Balafkan N., Tzoulis C., Muller B., Haugarvoll K., Tysnes O.B., Larsen J.P., Bindoff L.A. (2012). Number of CAG repeats in POLG1 and its association with Parkinson disease in the Norwegian population. Mitochondrion.

[bib0045] Baloyannis S.J., Costa V., Baloyannis I.S. (2006). Morphological alterations of the synapses in the locus coeruleus in Parkinson's disease. J. Neurol. Sci..

[bib0050] Bedford L., Hay D., Devoy A., Paine S., Powe D.G., Seth R., Gray T., Topham I., Fone K., Rezvani N., Mee M., Soane T., Layfield R., Sheppard P.W., Ebendal T., Usoskin D., Lowe J., Mayer R.J. (2008). Depletion of 26S proteasomes in mouse brain neurons causes neurodegeneration and Lewy-like inclusions resembling human pale bodies. J. Neurosci..

[bib0055] Bender A., Krishnan K.J., Morris C.M., Taylor G.A., Reeve A.K., Perry R.H., Jaros E., Hersheson J.S., Betts J., Klopstock T., Taylor R.W., Turnbull D.M. (2006). High levels of mitochondrial DNA deletions in substantia nigra neurons in aging and Parkinson disease. Nat. Genet..

[bib0060] Bender A., Schwarzkopf R.M., McMillan A., Krishnan K.J., Rieder G., Neumann M., Elstner M., Turnbull D.M., Klopstock T. (2008). Dopaminergic midbrain neurons are the prime target for mitochondrial DNA deletions. J. Neurol..

[bib0065] Bendor J.T., Logan T.P., Edwards R.H. (2013). The function of alpha-synuclein. Neuron.

[bib0070] Betarbet R., Sherer T.B., MacKenzie G., Garcia-Osuna M., Panov A.V., Greenamyre J.T. (2000). Chronic systemic pesticide exposure reproduces features of Parkinson's disease. Nat. Neurosci..

[bib0075] Betts-Henderson J., Jaros E., Krishnan K.J., Perry R.H., Reeve A.K., Schaefer A.M., Taylor R.W., Turnbull D.M. (2009). Alpha-synuclein pathology and Parkinsonism associated with POLG1 mutations and multiple mitochondrial DNA deletions. Neuropathol. Appl. Neurobiol..

[bib0080] Bilgic B., Pfefferbaum A., Rohlfing T., Sullivan E.V., Adalsteinsson E. (2012). MRI estimates of brain iron concentration in normal aging using quantitative susceptibility mapping. Neuroimage.

[bib0085] Botella J.A., Bayersdorfer F., Schneuwly S. (2008). Superoxide dismutase overexpression protects dopaminergic neurons in a Drosophila model of Parkinson's disease. Neurobiol. Dis..

[bib0090] Braak H., Braak E., Yilmazer D., Schultz C., de Vos R.A., Jansen E.N. (1995). Nigral and extranigral pathology in Parkinson's disease. J. Neural Transm. Suppl..

[bib0095] Braak E., Sandmann-Keil D., Rub U., Gai W.P., de Vos R.A., Steur E.N., Arai K., Braak H. (2001). alpha-Synuclein immunopositive Parkinson's disease-related inclusion bodies in lower brain stem nuclei. Acta Neuropathol..

[bib0100] Braak H., Ghebremedhin E., Rub U., Bratzke H., Del Tredici K. (2004). Stages in the development of Parkinson's disease-related pathology. Cell Tissue Res..

[bib0105] Breydo L., Wu J.W., Uversky V.N. (2012). alpha-Synuclein misfolding and Parkinson's disease. Biochim. Biophys. Acta.

[bib0110] Buchman A.S., Shulman J.M., Nag S., Leurgans S.E., Arnold S.E., Morris M.C., Schneider J.A., Bennett D.A. (2012). Nigral pathology and Parkinsonian signs in elders without Parkinson disease. Ann. Neurol..

[bib0115] Cali T., Ottolini D., Negro A., Brini M. (2012). alpha-Synuclein controls mitochondrial calcium homeostasis by enhancing endoplasmic reticulum-mitochondria interactions. J. Biol. Chem..

[bib0120] Campbell G.R., Ziabreva I., Reeve A.K., Krishnan K.J., Reynolds R., Howell O., Lassmann H., Turnbull D.M., Mahad D.J. (2011). Mitochondrial DNA deletions and neurodegeneration in multiple sclerosis. Ann. Neurol..

[bib0125] Casali C., Bonifati V., Santorelli F.M., Casari G., Fortini D., Patrignani A., Fabbrini G., Carrozzo R., D’Amati G., Locuratolo N., Vanacore N., Damiano M., Pierallini A., Pierelli F., Amabile G.A., Meco G. (2001). Mitochondrial myopathy, Parkinsonism, and multiple mtDNA deletions in a Sephardic Jewish family. Neurology.

[bib0130] Chan C.S., Guzman J.N., Ilijic E., Mercer J.N., Rick C., Tkatch T., Meredith G.E., Surmeier D.J. (2007). ‘Rejuvenation’ protects neurons in mouse models of Parkinson's disease. Nature.

[bib0135] Chen Q., Thorpe J., Keller J.N. (2005). Alpha-synuclein alters proteasome function, protein synthesis, and stationary phase viability. J. Biol. Chem..

[bib0140] Cheng F., Vivacqua G., Yu S. (2011). The role of alpha-synuclein in neurotransmission and synaptic plasticity. J. Chem. Neuroanat..

[bib0145] Chiti F., Dobson C.M. (2006). Protein misfolding, functional amyloid, and human disease. Annu. Rev. Biochem..

[bib0150] Clark I.E., Dodson M.W., Jiang C., Cao J.H., Huh J.R., Seol J.H., Yoo S.J., Hay B.A., Guo M. (2006). Drosophila pink1 is required for mitochondrial function and interacts genetically with Parkin. Nature.

[bib0155] Corral-Debrinski M., Horton T., Lott M.T., Shoffner J.M., Beal M.F., Wallace D.C. (1992). Mitochondrial DNA deletions in human brain: regional variability and increase with advanced age. Nat. Genet..

[bib0160] Daugherty A., Raz N. (2013). Age-related differences in iron content of subcortical nuclei observed in vivo: a meta-analysis. Neuroimage.

[bib0165] Davidzon G., Greene P., Mancuso M., Klos K.J., Ahlskog J.E., Hirano M., DiMauro S. (2006). Early-onset familial Parkinsonism due to POLG mutations. Ann. Neurol..

[bib0170] de Brito O.M., Scorrano L. (2008). Mitofusin 2 tethers endoplasmic reticulum to mitochondria. Nature.

[bib0175] de Lau L.M., Breteler M.M. (2006). Epidemiology of Parkinson's disease. Lancet Neurol..

[bib0180] Del Tredici K., Braak H. (2012). Lewy pathology and neurodegeneration in premotor Parkinson's disease. Mov. Disord..

[bib0185] Devi L., Raghavendran V., Prabhu B.M., Avadhani N.G., Anandatheerthavarada H.K. (2008). Mitochondrial import and accumulation of alpha-synuclein impair complex I in human dopaminergic neuronal cultures and Parkinson disease brain. J. Biol. Chem..

[bib0190] Dexter D.T., Wells F.R., Lees A.J., Agid F., Agid Y., Jenner P., Marsden C.D. (1989). Increased nigral iron content and alterations in other metal ions occurring in brain in Parkinson's disease. J. Neurochem..

[bib0195] Double K.L., Gerlach M., Schunemann V., Trautwein A.X., Zecca L., Gallorini M., Youdim M.B., Riederer P., Ben-Shachar D. (2003). Iron-binding characteristics of neuromelanin of the human substantia nigra. Biochem. Pharmacol..

[bib0200] Double K.L., Dedov V.N., Fedorow H., Kettle E., Halliday G.M., Garner B., Brunk U.T. (2008). The comparative biology of neuromelanin and lipofuscin in the human brain. Cell. Mol. Life Sci..

[bib0205] Dugger B.N., Murray M.E., Boeve B.F., Parisi J.E., Benarroch E.E., Ferman T.J., Dickson D.W. (2012). Neuropathological analysis of brainstem cholinergic and catecholaminergic nuclei in relation to rapid eye movement (REM) sleep behaviour disorder. Neuropathol. Appl. Neurobiol..

[bib0210] Eerola J., Luoma P.T., Peuralinna T., Scholz S., Paisan-Ruiz C., Suomalainen A., Singleton A.B., Tienari P.J. (2010). POLG1 polyglutamine tract variants associated with Parkinson's disease. Neurosci. Lett..

[bib0215] Ekstrand M.I., Terzioglu M., Galter D., Zhu S., Hofstetter C., Lindqvist E., Thams S., Bergstrand A., Hansson F.S., Trifunovic A., Hoffer B., Cullheim S., Mohammed A.H., Olson L., Larsson N.G. (2007). Progressive Parkinsonism in mice with respiratory-chain-deficient dopamine neurons. Proc. Natl. Acad. Sci. U.S.A..

[bib0220] Elson J.L., Samuels D.C., Turnbull D.M., Chinnery P.F. (2001). Random intracellular drift explains the clonal expansion of mitochondrial DNA mutations with age. Am. J. Hum. Genet..

[bib0225] Elstner M., Muller S.K., Leidolt L., Laub C., Krieg L., Schlaudraff F., Liss B., Morris C., Turnbull D.M., Masliah E., Prokisch H., Klopstock T., Bender A. (2011). Neuromelanin, neurotransmitter status and brainstem location determine the differential vulnerability of catecholaminergic neurons to mitochondrial DNA deletions. Mol. Brain.

[bib0230] Emmanouilidou E., Stefanis L., Vekrellis K. (2010). Cell-produced alpha-synuclein oligomers are targeted to, and impair, the 26S proteasome. Neurobiol. Aging.

[bib0235] Fearnley J.M., Lees A.J. (1991). Ageing and Parkinson's disease: substantia nigra regional selectivity. Brain.

[bib0240] Fedorow H., Halliday G.M., Rickert C.H., Gerlach M., Riederer P., Double K.L. (2006). Evidence for specific phases in the development of human neuromelanin. Neurobiol. Aging.

[bib0245] Friedman A., Galazka-Friedman J., Bauminger E.R. (2007). Iron as a trigger of neurodegeneration in Parkinson's disease. Hand. Clin. Neurol..

[bib0250] Fujiwara H., Hasegawa M., Dohmae N., Kawashima A., Masliah E., Goldberg M.S., Shen J., Takio K., Iwatsubo T. (2002). alpha-Synuclein is phosphorylated in synucleinopathy lesions. Nat. Cell Biol..

[bib0255] Gasser T., Hardy J., Mizuno Y. (2011). Milestones in PD genetics. Mov. Disord..

[bib0260] Gesi M., Soldani P., Giorgi F.S., Santinami A., Bonaccorsi I., Fornai F. (2000). The role of the locus coeruleus in the development of Parkinson's disease. Neurosci. Biobehav. Rev..

[bib0265] Giasson B.I., Duda J.E., Murray I.V., Chen Q., Souza J.M., Hurtig H.I., Ischiropoulos H., Trojanowski J.Q., Lee V.M. (2000). Oxidative damage linked to neurodegeneration by selective alpha-synuclein nitration in synucleinopathy lesions. Science.

[bib0270] Gibb W.R., Lees A.J. (1991). Anatomy, pigmentation, ventral and dorsal subpopulations of the substantia nigra, and differential cell death in Parkinson's disease. J. Neurol. Neurosurg. Psychiatry.

[bib0275] Goldberg J.A., Guzman J.N., Estep C.M., Ilijic E., Kondapalli J., Sanchez-Padilla J., Surmeier D.J. (2012). Calcium entry induces mitochondrial oxidant stress in vagal neurons at risk in Parkinson's disease. Nat. Neurosci..

[bib0280] Goo H.G., Jung M.K., Han S.S., Rhim H., Kang S. (2013). HtrA2/Omi deficiency causes damage and mutation of mitochondrial DNA. Biochim. Biophys. Acta.

[bib0285] Greene J.C., Whitworth A.J., Kuo I., Andrews L.A., Feany M.B., Pallanck L.J. (2003). Mitochondrial pathology and apoptotic muscle degeneration in Drosophila Parkin mutants. Proc. Natl. Acad. Sci. U.S.A..

[bib0290] Haacke E.M., Miao Y., Liu M., Habib C.A., Katkuri Y., Liu T., Yang Z., Lang Z., Hu J., Wu J. (2010). Correlation of putative iron content as represented by changes in R2* and phase with age in deep gray matter of healthy adults. J. Magn. Reson. Imaging.

[bib0295] Halliday G.M., Ophof A., Broe M., Jensen P.H., Kettle E., Fedorow H., Cartwright M.I., Griffiths F.M., Shepherd C.E., Double K.L. (2005). Alpha-synuclein redistributes to neuromelanin lipid in the substantia nigra early in Parkinson's disease. Brain.

[bib0300] Hara T., Nakamura K., Matsui M., Yamamoto A., Nakahara Y., Suzuki-Migishima R., Yokoyama M., Mishima K., Saito I., Okano H., Mizushima N. (2006). Suppression of basal autophagy in neural cells causes neurodegenerative disease in mice. Nature.

[bib0305] Hirsch E.C., Graybiel A.M., Duyckaerts C., Javoy-Agid F. (1987). Neuronal loss in the pedunculopontine tegmental nucleus in Parkinson disease and in progressive supranuclear palsy. Proc. Natl. Acad. Sci. U.S.A..

[bib0310] Horvath R., Kley R.A., Lochmuller H., Vorgerd M. (2007). Parkinson syndrome, neuropathy, and myopathy caused by the mutation A8344G (MERRF) in tRNALys. Neurology.

[bib0315] Hudson G., Schaefer A.M., Taylor R.W., Tiangyou W., Gibson A., Venables G., Griffiths P., Burn D.J., Turnbull D.M., Chinnery P.F. (2007). Mutation of the linker region of the polymerase gamma-1 (POLG1) gene associated with progressive external ophthalmoplegia and Parkinsonism. Arch. Neurol..

[bib0320] Hudson G., Nalls M., Evans J.R., Breen D.P., Winder-Rhodes S., Morrison K.E., Morris H.R., Williams-Gray C.H., Barker R.A., Singleton A.B., Hardy J., Wood N.E., Burn D.J., Chinnery P.F. (2013). Two-stage association study and meta-analysis of mitochondrial DNA variants in Parkinson disease. Neurology.

[bib0325] Huerta C., Castro M.G., Coto E., Blazquez M., Ribacoba R., Guisasola L.M., Salvador C., Martinez C., Lahoz C.H., Alvarez V. (2005). Mitochondrial DNA polymorphisms and risk of Parkinson's disease in Spanish population. J. Neurol. Sci..

[bib0330] Itoh K., Weis S., Mehraein P., Muller-Hocker J. (1996). Cytochrome c oxidase defects of the human substantia nigra in normal aging. Neurobiol. Aging.

[bib0335] Jana N.R. (2012). Protein homeostasis and aging: role of ubiquitin protein ligases. Neurochem. Int..

[bib0340] Kalia L.V., Kalia S.K., McLean P.J., Lozano A.M., Lang A.E. (2013). alpha-Synuclein oligomers and clinical implications for Parkinson disease. Ann. Neurol..

[bib0345] Kastner A., Hirsch E.C., Lejeune O., Javoy-Agid F., Rascol O., Agid Y. (1992). Is the vulnerability of neurons in the substantia nigra of patients with Parkinson's disease related to their neuromelanin content?. J. Neurochem..

[bib0350] Khaliq Z.M., Bean B.P. (2010). Pacemaking in dopaminergic ventral tegmental area neurons: depolarizing drive from background and voltage-dependent sodium conductances. J. Neurosci..

[bib0355] Komatsu M., Waguri S., Chiba T., Murata S., Iwata J., Tanida I., Ueno T., Koike M., Uchiyama Y., Kominami E., Tanaka K. (2006). Loss of autophagy in the central nervous system causes neurodegeneration in mice. Nature.

[bib0360] Kraytsberg Y., Kudryavtseva E., McKee A.C., Geula C., Kowall N.W., Khrapko K. (2006). Mitochondrial DNA deletions are abundant and cause functional impairment in aged human substantia nigra neurons. Nat. Genet..

[bib0365] Krishnan K.J., Reeve A.K., Samuels D.C., Chinnery P.F., Blackwood J.K., Taylor R.W., Wanrooij S., Spelbrink J.N., Lightowlers R.N., Turnbull D.M. (2008). What causes mitochondrial DNA deletions in human cells?. Nat. Genet..

[bib0370] Krishnan K.J., Ratnaike T.E., De Gruyter H.L., Jaros E., Turnbull D.M. (2012). Mitochondrial DNA deletions cause the biochemical defect observed in Alzheimer's disease. Neurobiol. Aging.

[bib0375] Langston J.W., Ballard P.A. (1983). Parkinson's disease in a chemist working with 1-methyl-4-phenyl-1,2,5,6-tetrahydropyridine. N. Engl. J. Med..

[bib0380] Langston J.W., Ballard P., Tetrud J.W., Irwin I. (1983). Chronic Parkinsonism in humans due to a product of meperidine-analog synthesis. Science.

[bib0385] Latsoudis H., Spanaki C., Chlouverakis G., Plaitakis A. (2008). Mitochondrial DNA polymorphisms and haplogroups in Parkinson's disease and control individuals with a similar genetic background. J. Hum. Genet..

[bib0390] Lax N.Z., Hepplewhite P.D., Reeve A.K., Nesbitt V., McFarland R., Jaros E., Taylor R.W., Turnbull D.M. (2012). Cerebellar ataxia in patients with mitochondrial DNA disease: a molecular clinicopathological study. J. Neuropathol. Exp. Neurol..

[bib0395] Lee S., Sterky F.H., Mourier A., Terzioglu M., Cullheim S., Olson L., Larsson N.G. (2012). Mitofusin 2 is necessary for striatal axonal projections of midbrain dopamine neurons. Hum. Mol. Genet..

[bib0400] Leverenz J.B., Hamilton R., Tsuang D.W., Schantz A., Vavrek D., Larson E.B., Kukull W.A., Lopez O., Galasko D., Masliah E., Kaye J., Woltjer R., Clark C., Trojanowski J.Q., Montine T.J. (2008). Empiric refinement of the pathologic assessment of Lewy-related pathology in the dementia patient. Brain Pathol..

[bib0405] Li X.J., Li S. (2011). Proteasomal dysfunction in aging and Huntington disease. Neurobiol. Dis..

[bib0410] Lowe J. (2008). Neuropathology of dementia with Lewy bodies. Handbook Clin. Neurol..

[bib0415] Luoma P., Melberg A., Rinne J.O., Kaukonen J.A., Nupponen N.N., Chalmers R.M., Oldfors A., Rautakorpi I., Peltonen L., Majamaa K., Somer H., Suomalainen A. (2004). Parkinsonism, premature menopause, and mitochondrial DNA polymerase gamma mutations: clinical and molecular genetic study. Lancet.

[bib0420] Ma S.Y., Ciliax B.J., Stebbins G., Jaffar S., Joyce J.N., Cochran E.J., Kordower J.H., Mash D.C., Levey A.I., Mufson E.J. (1999). Dopamine transporter-immunoreactive neurons decrease with age in the human substantia nigra. J. Comp. Neurol..

[bib0425] Ma S.Y., Roytt M., Collan Y., Rinne J.O. (1999). Unbiased morphometrical measurements show loss of pigmented nigral neurones with ageing. Neuropathol. Appl. Neurobiol..

[bib0430] Ma X., Liu H., Foyil S.R., Godar R.J., Weinheimer C.J., Hill J.A., Diwan A. (2012). Impaired autophagosome clearance contributes to cardiomyocyte death in ischemia/reperfusion injury. Circulation.

[bib0435] Maingay M., Romero-Ramos M., Carta M., Kirik D. (2006). Ventral tegmental area dopamine neurons are resistant to human mutant alpha-synuclein overexpression. Neurobiol. Dis..

[bib0440] Mancuso M., Filosto M., Oh S.J., DiMauro S. (2004). A novel polymerase gamma mutation in a family with ophthalmoplegia, neuropathy, and Parkinsonism. Arch. Neurol..

[bib0445] Martin L.J., Pan Y., Price A.C., Sterling W., Copeland N.G., Jenkins N.A., Price D.L., Lee M.K. (2006). Parkinson's disease alpha-synuclein transgenic mice develop neuronal mitochondrial degeneration and cell death. J. Neurosci..

[bib0450] McNaught K.S., Jenner P. (2001). Proteasomal function is impaired in substantia nigra in Parkinson's disease. Neurosci. Lett..

[bib0455] McNaught K.S., Belizaire R., Isacson O., Jenner P., Olanow C.W. (2003). Altered proteasomal function in sporadic Parkinson's disease. Exp. Neurol..

[bib0460] Mehta P., Mellick G.D., Rowe D.B., Halliday G.M., Jones M.M., Manwaring N., Vandebona H., Silburn P.A., Wang J.J., Mitchell P., Sue C.M. (2009). Mitochondrial DNA haplogroups J and K are not protective for Parkinson's disease in the Australian community. Mov. Disord..

[bib0465] Miller K.E., Sheetz M.P. (2004). Axonal mitochondrial transport and potential are correlated. J. Cell Sci..

[bib0470] Mortiboys H., Johansen K.K., Aasly J.O., Bandmann O. (2010). Mitochondrial impairment in patients with Parkinson disease with the G2019S mutation in LRRK2. Neurology.

[bib0475] Murphy D.D., Rueter S.M., Trojanowski J.Q., Lee V.M. (2000). Synucleins are developmentally expressed, and alpha-synuclein regulates the size of the presynaptic vesicular pool in primary hippocampal neurons. J. Neurosci..

[bib0480] Nakamura K., Nemani V.M., Azarbal F., Skibinski G., Levy J.M., Egami K., Munishkina L., Zhang J., Gardner B., Wakabayashi J., Sesaki H., Cheng Y., Finkbeiner S., Nussbaum R.L., Masliah E., Edwards R.H. (2011). Direct membrane association drives mitochondrial fission by the Parkinson disease-associated protein alpha-synuclein. J. Biol. Chem..

[bib0485] Nussbaum R.L., Ellis C.E. (2003). Alzheimer's disease and Parkinson's disease. N. Engl. J. Med..

[bib0490] Ottolini D., Cali T., Negro A., Brini M. (2013). The Parkinson disease-related protein DJ-1 counteracts mitochondrial impairment induced by the tumour suppressor protein p53 by enhancing endoplasmic reticulum-mitochondria tethering. Hum. Mol. Genet..

[bib0495] Pahapill P.A., Lozano A.M. (2000). The pedunculopontine nucleus and Parkinson's disease. Brain.

[bib0500] Pakkenberg B., Gundersen H.J. (1997). Neocortical neuron number in humans: effect of sex and age. J. Comp. Neurol..

[bib0505] Parihar M.S., Parihar A., Fujita M., Hashimoto M., Ghafourifar P. (2008). Mitochondrial association of alpha-synuclein causes oxidative stress. Cell. Mol. Life Sci..

[bib0510] Parihar M.S., Parihar A., Fujita M., Hashimoto M., Ghafourifar P. (2009). Alpha-synuclein overexpression and aggregation exacerbates impairment of mitochondrial functions by augmenting oxidative stress in human neuroblastoma cells. Int. J. Biochem. Cell Biol..

[bib0515] Park J., Kim S.Y., Cha G.H., Lee S.B., Kim S., Chung J. (2005). Drosophila DJ-1 mutants show oxidative stress-sensitive locomotive dysfunction. Gene.

[bib0520] Park J., Lee G., Chung J. (2009). The PINK1-Parkin pathway is involved in the regulation of mitochondrial remodeling process. Biochem. Biophys. Res. Commun..

[bib0525] Perier C., Bender A., Garcia-Arumi E., Melia M.J., Bove J., Laub C., Klopstock T., Elstner M., Mounsey R.B., Teismann P., Prolla T., Andreu A.L., Vila M. (2013). Accumulation of mitochondrial DNA deletions within dopaminergic neurons triggers neuroprotective mechanisms. Brain.

[bib0530] Pickrell A.M., Pinto M., Hida A., Moraes C.T. (2011). Striatal dysfunctions associated with mitochondrial DNA damage in dopaminergic neurons in a mouse model of Parkinson's disease. J. Neurosci..

[bib0535] Pissadaki E.K., Bolam J.P. (2013). The energy cost of action potential propagation in dopamine neurons: clues to susceptibility in Parkinson's disease. Front. Comput. Neurosci..

[bib0540] Pyle A., Foltynie T., Tiangyou W., Lambert C., Keers S.M., Allcock L.M., Davison J., Lewis S.J., Perry R.H., Barker R., Burn D.J., Chinnery P.F. (2005). Mitochondrial DNA haplogroup cluster UKJT reduces the risk of PD. Ann. Neurol..

[bib0545] Reddy P.H., Shirendeb U.P. (2012). Mutant huntingtin, abnormal mitochondrial dynamics, defective axonal transport of mitochondria, and selective synaptic degeneration in Huntington's disease. Biochim. Biophys. Acta.

[bib0550] Reddy P.H., Tripathi R., Troung Q., Tirumala K., Reddy T.P., Anekonda V., Shirendeb U.P., Calkins M.J., Reddy A.P., Mao P., Manczak M. (2012). Abnormal mitochondrial dynamics and synaptic degeneration as early events in Alzheimer's disease: implications to mitochondria-targeted antioxidant therapeutics. Biochim. Biophys. Acta.

[bib0555] Reeve A.K., Krishnan K.J., Taylor G., Elson J.L., Bender A., Taylor R.W., Morris C.M., Turnbull D.M. (2009). The low abundance of clonally expanded mitochondrial DNA point mutations in aged substantia nigra neurons. Aging Cell.

[bib0560] Reeve A.K., Park T.K., Jaros E., Campbell G.R., Lax N.Z., Hepplewhite P.D., Krishnan K.J., Elson J.L., Morris C.M., McKeith I.G., Turnbull D.M. (2012). Relationship between mitochondria and alpha-synuclein: a study of single substantia nigra neurons. Arch. Neurol..

[bib0565] Reeve A., Meagher M., Lax N., Simcox E., Hepplewhite P., Jaros E., Turnbull D. (2013). The impact of pathogenic mitochondrial DNA mutations on substantia nigra neurons. J. Neurosci..

[bib0570] Rubinsztein D.C., Marino G., Kroemer G. (2011). Autophagy and aging. Cell.

[bib0575] Rudow G., O’Brien R., Savonenko A.V., Resnick S.M., Zonderman A.B., Pletnikova O., Marsh L., Dawson T.M., Crain B.J., West M.J., Troncoso J.C. (2008). Morphometry of the human substantia nigra in ageing and Parkinson's disease. Acta Neuropathol..

[bib0580] Schapira A.H., Cooper J.M., Dexter D., Jenner P., Clark J.B., Marsden C.D. (1989). Mitochondrial complex I deficiency in Parkinson's disease. Lancet.

[bib0585] Schapira A.H., Cooper J.M., Dexter D., Clark J.B., Jenner P., Marsden C.D. (1990). Mitochondrial complex I deficiency in Parkinson's disease. J. Neurochem..

[bib0590] Shima T., Sarna T., Swartz H.M., Stroppolo A., Gerbasi R., Zecca L. (1997). Binding of iron to neuromelanin of human substantia nigra and synthetic melanin: an electron paramagnetic resonance spectroscopy study. Free Radic. Biol. Med..

[bib0595] Siciliano G., Mancuso M., Ceravolo R., Lombardi V., Iudice A., Bonuccelli U. (2001). Mitochondrial DNA rearrangements in young onset Parkinsonism: two case reports. J. Neurol. Neurosurg. Psychiatry.

[bib0600] Simon D.K., Pulst S.M., Sutton J.P., Browne S.E., Beal M.F., Johns D.R. (1999). Familial multisystem degeneration with Parkinsonism associated with the 11778 mitochondrial DNA mutation. Neurology.

[bib0605] Sofic E., Paulus W., Jellinger K., Riederer P., Youdim M.B. (1991). Selective increase of iron in substantia nigra zona compacta of Parkinsonian brains. J. Neurochem..

[bib0610] Song L., Shan Y., Lloyd K.C., Cortopassi G.A. (2012). Mutant Twinkle increases dopaminergic neurodegeneration, mtDNA deletions and modulates Parkin expression. Hum. Mol. Genet..

[bib0615] Soong N.W., Hinton D.R., Cortopassi G., Arnheim N. (1992). Mosaicism for a specific somatic mitochondrial DNA mutation in adult human brain. Nat. Genet..

[bib0620] Sterky F.H., Lee S., Wibom R., Olson L., Larsson N.G. (2011). From the cover: impaired mitochondrial transport and Parkin-independent degeneration of respiratory chain-deficient dopamine neurons in vivo. Proc. Natl. Acad. Sci. U.S.A..

[bib0625] Sulzer D. (2007). Multiple hit hypotheses for dopamine neuron loss in Parkinson's disease. Trends Neurosci..

[bib0630] Surmeier D.J., Sulzer D. (2013). The pathology roadmap in Parkinson disease. Prion.

[bib0635] Tanaka Y., Engelender S., Igarashi S., Rao R.K., Wanner T., Tanzi R.E., Sawa A.V.L.D, Dawson T.M., Ross C.A. (2001). Inducible expression of mutant alpha-synuclein decreases proteasome activity and increases sensitivity to mitochondria-dependent apoptosis. Hum. Mol. Genet..

[bib0640] Taylor R.W., Barron M.J., Borthwick G.M., Gospel A., Chinnery P.F., Samuels D.C., Taylor G.A., Plusa S.M., Meddham S.J., Greaves L.C., Kirkwood B.L., Turnbull D.M. (2003). Mitochondrial mutations in human colonic crypt stem cells. J. Clin. Invest..

[bib0645] Thomas K.J., McCoy M.K., Blackinton J., Beilina A., van der Brug M., Sandebring A., Miller D., Maric D., Cedazo-Minguez A., Cookson M.R. (2010). DJ-1 acts in parallel to the PINK1/parkin pathway to control mitochondrial function and autophagy. Hum. Mol. Genet..

[bib0650] Trevelyan A.J., Kirby D.M., Smulders-Srinivasan T.K., Nooteboom M., Acin-Perez R., Enriquez J.A., Whittington M.A., Lightowlers R.N., Turnbull D.M. (2010). Mitochondrial DNA mutations affect calcium handling in differentiated neurons. Brain.

[bib0655] Trifunovic A., Wredenberg A., Falkenberg M., Spelbrink J.N., Rovio A.T., Bruder C.E., Bohlooly Y.M., Gidlof S., Oldfors A., Wibom R., Tornell J., Jacobs H.T., Larsson N.G. (2004). Premature ageing in mice expressing defective mitochondrial DNA polymerase. Nature.

[bib0660] Twig G., Elorza A., Molina A.J., Mohamed H., Wikstrom J.D., Walzer G., Stiles L., Haigh S.E., Katz S., Las G., Alroy J., Wu M., Py B.F., Yuan J., Deeney J.T., Corkey B.E., Shirihai O.S. (2008). Fission and selective fusion govern mitochondrial segregation and elimination by autophagy. EMBO J..

[bib0665] Uchikado H., Lin W.L., DeLucia M.W., Dickson D.W. (2006). Alzheimer disease with amygdala Lewy bodies: a distinct form of alpha-synucleinopathy. J. Neuropathol. Exp. Neurol..

[bib0670] van der Walt J.M., Nicodemus K.K., Martin E.R., Scott W.K., Nance M.A., Watts R.L., Hubble J.P., Haines J.L., Koller W.C., Lyons K., Pahwa R., Stern M.B., Colcher A., Hiner B.C., Jankovic J., Ondo W.G., Allen F.H., Goetz C.G., Small G.W., Mastaglia F., Stajich J.M., McLaurin A.C., Middleton L.T., Scott B.L., Schmechel D.E., Pericak-Vance M.A., Vance J.M. (2003). Mitochondrial polymorphisms significantly reduce the risk of Parkinson disease. Am. J. Hum. Genet..

[bib0675] Verburg J., Hollenbeck P.J. (2008). Mitochondrial membrane potential in axons increases with local nerve growth factor or semaphorin signaling. J. Neurosci..

[bib0680] Vincow E.S., Merrihew G., Thomas R.E., Shulman N.J., Beyer R.P., MacCoss M.J., Pallanck L.J. (2013). The PINK1-Parkin pathway promotes both mitophagy and selective respiratory chain turnover in vivo. Proc. Natl. Acad. Sci. U.S.A..

[bib0685] Vogiatzi T., Xilouri M., Vekrellis K., Stefanis L. (2008). Wild type alpha-synuclein is degraded by chaperone-mediated autophagy and macroautophagy in neuronal cells. J. Biol. Chem..

[bib0690] Wang X., Petrie T.G., Liu Y., Liu J., Fujioka H., Zhu X. (2012). Parkinson's disease-associated DJ-1 mutations impair mitochondrial dynamics and cause mitochondrial dysfunction. J. Neurochem..

[bib0695] Weihofen A., Thomas K.J., Ostaszewski B.L., Cookson M.R., Selkoe D.J. (2009). Pink1 forms a multiprotein complex with Miro and Milton, linking Pink1 function to mitochondrial trafficking. Biochemistry.

[bib0700] Wood-Kaczmar A., Gandhi S., Wood N.W. (2006). Understanding the molecular causes of Parkinson's disease. Trends Mol. Med..

[bib0705] Zecca L., Tampellini D., Gerlach M., Riederer P., Fariello R.G., Sulzer D. (2001). Substantia nigra neuromelanin: structure, synthesis, and molecular behaviour. Mol. Pathol..

[bib0710] Zhou C., Zhong W., Zhou J., Sheng F., Fang Z., Wei Y., Chen Y., Deng X., Xia B., Lin J. (2012). Monitoring autophagic flux by an improved tandem fluorescent-tagged LC3 (mTagRFP-mWasabi-LC3) reveals that high-dose rapamycin impairs autophagic flux in cancer cells. Autophagy.

